# Highlighting a curricular need: Uncertainty, COVID‐19, and health systems science

**DOI:** 10.1002/hsr2.363

**Published:** 2021-08-31

**Authors:** Dimitrios Papanagnou, Rebecca Jaffe, Deborah Ziring

**Affiliations:** ^1^ Department of Emergency Medicine Sidney Kimmel Medical College at Thomas Jefferson University Philadelphia Pennsylvania USA; ^2^ 2020 Macy Faculty Scholar through the Josiah Macy, Jr. Foundation New York New York USA; ^3^ Department of Medicine Sidney Kimmel Medical College at Thomas Jefferson University Philadelphia Pennsylvania USA

## 1 PERSPECTIVE

Healthcare is increasingly complex; with increased complexity comes increased uncertainty. Historically, however, most medical practice and medical education occur in the context of certainty. The coronavirus disease 2019 (COVID‐19) pandemic has illuminated the gap in training students in the health professions to function in environments of both certainty and uncertainty. Medical students are typically presented with facts, associations, and algorithms, and are later assessed through methods that either has a single correct answer or a series of acceptable answers with a single meaning (eg, multiple‐choice examinations and objective structured clinical examinations).^1^ The transition from the classroom to the clinical learning environment poses medical students with significant struggles. These may include struggles with diagnosis, management, communication, patient care, and care coordination. To date, formal training and assessment to enhance comfort with and management of uncertainty in clinical practice are lacking in many training programs.^1^ A curriculum in Health Systems Science (HSS) can specifically acknowledge and address the uncertainty intrinsic to modern medical care and, in doing so, better prepare students for the transition to the clinical environment, the intrinsic complexity of the healthcare system, and the uncertainty of professional clinical practice.

### 1.1 Uncertainty

Uncertainty in clinical medicine affects all parts of clinical practice, from diagnosis to treatment decisions.^2^ Lee et al define uncertainty as “the dynamic, subjective perception of not knowing what to think, feel, or do.”^3^ To this effect, three core dimensions of uncertainty in clinical practice are described: the sources of uncertainty, the subjective nature of uncertainty, and responses to uncertainty.^3^ The most common dimension in defining uncertainty is its source, which may stem from knowledge, relationships, and complex systems.^3^ The cognitive impact these sources of uncertainty have in practitioners, in turn, influences their respective responses and actions.^3^

Students ill‐equipped to address uncertainty in the clinical environment can undergo cognitive dissonance,^4^ diminished self‐efficacy, and erosion of empathy.^5^ Uncertainty can lead to maladaptive perfectionism and burnout.^6^, ^7^ The traditional emphasis on linear thinking in medical school can thwart creative problem‐solving approaches, alternative perspectives, and the ability to calibrate for uncertainty^5^—all of which are tools needed to thrive in today's clinical learning and working environment. Personal perspectives on uncertainty have been found to impact patient communication,^7^ decision‐making ability,^8^ resource utilization,^7^ and attitudes toward groups of patients.^9^ Physicians' anxiety toward clinical uncertainty is associated with increased cost of care, as well as a reluctance to fully disclose information to patients.^7^ Studies have even suggested that tolerance of uncertainty impacts students' willingness to work with underserved communities, and influences how providers address pain management during times of diagnostic ambiguity.^6^, ^9^, ^10^

Intentional training in uncertainty is important not only because of the increasing complexity of healthcare and its associated systems but also because of the way access to information has changed personal relationships with uncertainty. In a simple model of uncertainty, the Johari window defines domains in a two‐by‐two table of “known” or “unknown” to self, and “known” or “unknown” to others.^11^ Individuals experience their own uncertainty when something is unknown to them and either known or unknown to others. In a world of constant access to information at our fingertips, individuals have less exposure to situations where information is unknown or unknowable to them and known to others—take, for example, the use of navigation applications (apps) utilizing global positioning system (GPS) services on smartphone devices. This decrease in experience managing day‐to‐day uncertainty may decrease skill in managing the less common type of uncertainty where information, or truth, is unknown to self, as well as to others.

### 1.2 The clinical environment

The clinical learning and working environment are intrinsically uncertain, and the transition into this workplace is a point where students frequently struggle. First and foremost, the work is complex. Students are introduced to tools they have never encountered before, such as the electronic health record (EHR), which differs from institution to institution or may change several times at a single institution. New members are continuously added to teams. Team roles are in constant evolution, now including community health workers, acute care managers, and patient navigators. Teams are constantly in flux, with new members creating dynamic expectations and interactions. The practice milieu and overarching system of healthcare are also subject to change, as seen by the impact the Affordable Care Act has had on health delivery in the United States.^12^

The addition of learners (ie, students, residents, fellows, and allied health professionals) into the working environment only adds to this complexity. Multiple outcomes of interest—clinical and educational—compete for priority. While frameworks characterizing the clinical learning environment exist to describe its interrelated personal, social, organizational, physical, and virtual workplace components, each of which directly links to the broader health system,^13^, ^14^ the clinical environment is challenged by role clarity, the changing nature of teams, adequacy of learner supervision, and learner mistrust. It is often difficult for clinical teams immersed in the clinical learning environment (CLE) to make informed decisions during instances of heightened complexity, when the relationship between cause and effect is blurred, and when there are minimal causal loops to guide teams through the uncertainty. The recent COVID‐19 pandemic has greatly magnified the complexity intrinsic to the CLE, highlighting the need for formal training surrounding times of uncertainty.

### 1.3 The COVID‐19 pandemic

The COVID‐19 pandemic has illustrated how critical it is for physicians to function at a high level during times of uncertainty. Throughout the early stages of the pandemic, and as the demand for health services increased exponentially across health systems, clinical providers were forced to adapt and re‐adapt to rapidly evolving guidelines and settings.^15^ Correct answers were not immediately available. Shortages of healthcare professionals, adequate access to personal protective equipment (PPE), and the urgent need for training clinical teams on infection control and prevention exemplified instances in which volatility, uncertainty, complexity, and ambiguity challenged CLEs and health systems together.^16^ There was simply no time for clinical teams to step back and gain an understanding of the “big picture,” an overarching goal for systems thinking.^17^ The pace of change and progress outstripped the ability to take a purposeful and analytic approach. Providers, leaders, and learners were forced to act and react to a status quo that evolved and morphed daily. There was no stable state. Instead, individuals with the necessary aptitude to work in conditions of high uncertainty and chaos had to create local networks to learn, communicate, stabilize, and continuously adapt.

While most junior learners were removed from the clinical environment in the early days of the pandemic for safety and PPE preservation, this experience exemplified the need for skills in managing workplace disruption and uncertainty. Now, as junior learners re‐enter the CLE, it is clear that the pandemic accentuated several of the challenges that typically accompany the transition into this type of working environment. Several of these challenges include:

How do students contribute to the care of patients they are not allowed to assess in person?

How do learners manage a disease process they have never been formally taught to manage, especially in the face of rapidly changing evidence and guidelines, and variable expertise among supervising faculty?

How does one manage a clinical situation when personal risk for infection is unclear?

How should students manage themselves when:

1. their respective supervisor does not know an answer?
2. their role is suddenly unclear?
3. duration of training changes dramatically?

### 1.4 Making sense amidst uncertainty and complexity

It is essential that curricula in health professions education help students make sense of—and take action amidst—the heightened uncertainty they will encounter in their practice. In clinical situations that change dynamically and do not obey causal laws, where teams cannot predict the results of their actions, much like the early stages of the pandemic, teams will require a framework to guide their ability to learn together within the context of uncertainty and collaboratively develop their own solutions as they probe the system, make sense of it, and learn from it before they respond. To an extent, this is a practice that is already routinely observed in quality improvement patient safety initiatives that leverage iterative PDSA (Plan, Do, Study, Act) cycles of improvement. Such a framework should consider the individuals and teams within specific clinical contexts, and provide them with the respective scaffolding to make sense of their experiences as they work through uncertainty. The Cynefin framework, first introduced by David Snowden, represents such a framework for categorizing issues and strategies.^18^

**FIGURE 1:**
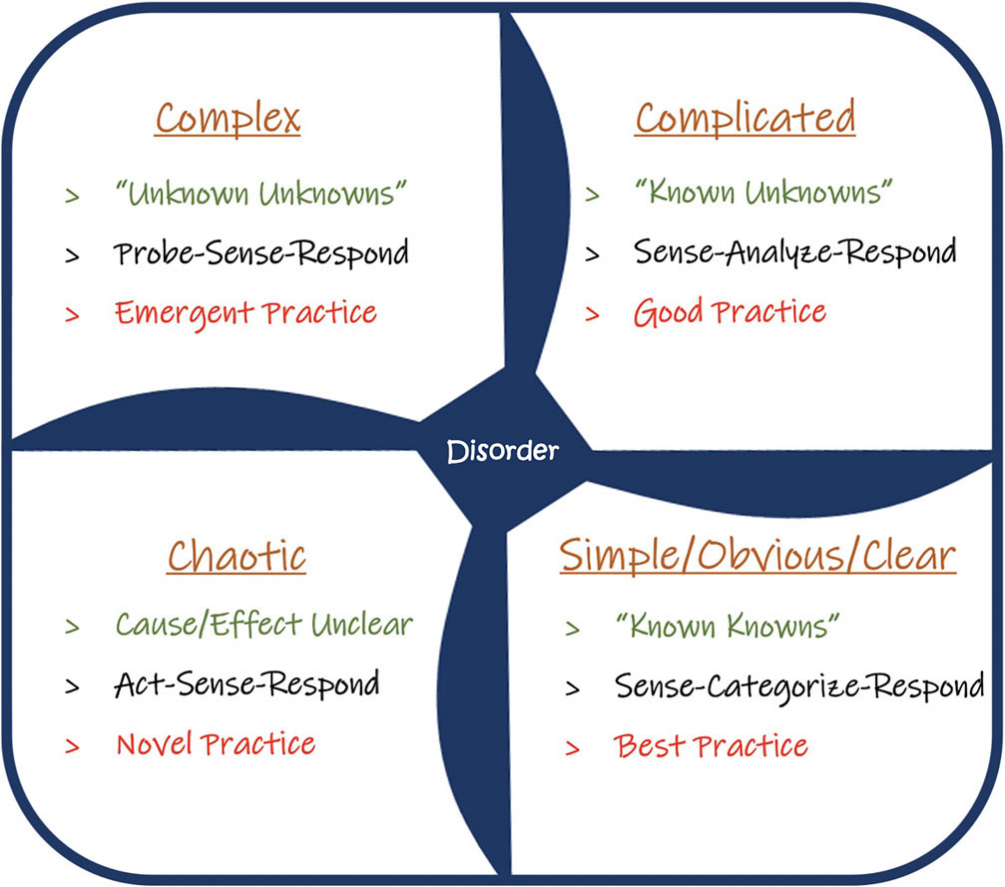
The Cynefin framework

The Cynefin framework is a conceptual framework that can aid in decision‐making during uncertain times and draws upon research from complexity science. The framework offers specific decision‐making contexts (or domains) in which individuals and/or teams of individuals can make sense of their experiences. Applying this framework to the uncertainty in the CLE, issues facing clinical teams are sorted into five unique contexts defined by the nature of the relationship between cause and effect.^18^ Four of these domains (ie, simple, complicated, complex, and chaotic) require clinicians and teams to diagnose situations and act in ways that are contextually appropriate (Figure 1).^18^ The two domains on the right represent the “ordered” domains of ‘simple’ and ‘complicated’—with clear links between cause and effect and where correct answers can be determined based on data and facts. The two domains on the left represent the “unordered” domains of “complex” and “chaos”—with no apparent cause and effect relationship, where emerging patterns inform future actions.^18^ A fifth domain, “disorder”, describes instances in which it is unclear, which of the four contexts is predominant. For clinical teams working in a complex system, such as the CLE during the time of COVID‐19, team members may not be aware of what they do not know. Teams must iteratively interact and experiment with the system to determine general patterns before a definitive answer, or even causality, becomes apparent.

There has been some integration of complexity science into health professions education. For example, Noyer et al investigated the influence of perceived task complexity on the diagnostic reasoning of osteopathy students and found that when prompted to perceive a case as complex, student reliance on analytical approaches increased at the expense of non‐analytical reasoning.^19^ The authors posit that to reduce cognitive load and avoid associated diagnostic error, however, strategies should be adopted that contextualize learning^19^; the Cynefin framework may represent such a strategy.

Aside from the integration of complexity science into health professions education, opportunities for curricular development that prepare students for this uncertainty exist. Incorporation of the liberal arts, humanities programs, and patient‐centered narratives into formal curriculum has been shown to improve students' abilities to think laterally.^20^, ^21^ Discussing the “philosophy of medicine” within clinical coursework can provide trainees with the familiarity to appraise and describe the uncertainty they are experiencing.^2^ The deliberate practice of communicating diagnostic uncertainty to patients, when a diagnosis to explain patients' symptoms is unknown, can prepare trainees for challenging, emotionally charged conversations.^22^, ^23^ These conversations can be embedded into objective structured clinical encounters (OSCEs) that are focused on communication training.^24^ Similarly, coursework in HSS is uniquely poised to introduce students to this uncertainty.

### 1.5 A curriculum in uncertainty: An opportunity for HSS

HSS is uniquely positioned to introduce medical students to the uncertainty that exists in clinical practice and as early as Day 1 of their training. HSS can borrow elements of the Cynefin framework and equip learners with skills in problem‐solving, such as being able to facilitate open, interactive communication that can help share ideas, such as critical incident debriefings, or generate and ask higher‐order, divergent questions (eg, “what if we” or “how might we” questions) during times of uncertainty. Medical schools have increasingly integrated HSS into their respective formal curricula to better prepare trainees to thrive in today's healthcare system. Described as the third educational pillar that complements the basic and clinical sciences, HSS provides a framework of competencies related to value‐based care, population health, interprofessional collaboration, health system improvement, and systems thinking.^25^ Reconciling the uncertainty that is part of clinical practice is not its own class or field of study; in actuality, it is a theme integrated across these HSS competencies.

A curriculum in HSS can provide students with tools to prepare them for the uncertainty and complexity of the clinical practice environment. Students will be immersed in situations where they will have to apply clinical reasoning skills and act with confidence in ambiguous situations.^26^ Students should have the opportunity to openly discuss, reconstruct, and redefine their understanding of clinical problems employing the tools and resources that will be immediately available to them.^20^ An understanding of HSS can equip students with the ability to appraise and/or diagnose the uncertainty they may encounter in clinical practice, which may be helpful in identifying next steps and strategies (ie, is a specific course of action in a specific clinical context a “knowable” or “unknowable” uncertainty).^27^ Knowledge gaps, for example, could be addressed through referencing the literature or consulting with team members, while other forms of uncertainty (eg, personal or conceptual) would require a more individualized approach.^27^

A curriculum in HSS exposes students to cognitive science and raises their awareness of patterns that allow them to think and act quickly during times of uncertainty.^27^ Conversations would focus on providers' craving for a sense of certainty, which often leads to premature closure and misdiagnosis.^27^ Similarly, and as a direct lesson of the pandemic, students require an understanding of best practices in teamwork, communication, and facilitating debriefing conversations with team members. Training in uncertainty could also improve patient‐centered care. For example, at least one‐third of patients are discharged from the emergency department without a diagnosis, and more than 40% of trainees encountered challenges with discharging patients with diagnostic uncertainty, with >50% of trainees reporting a strong desire for additional training in how to facilitate these conversations.^22^, ^23^ By discussing uncertainty with patients, whether about diagnosis, treatment, management, or prognosis, providers can navigate uncertainty through shared decision‐making.^27^

To address the web of interdependent and interrelated parts of the clinical environment, HSS has leveraged systems thinking as an approach to better understand how health systems work and how to navigate the complex adaptive challenges they pose.^17^ Given the complex nature of health systems, however, a curriculum in HSS can supplement a systems‐thinking approach with frameworks grounded in complexity science (eg, the Cynefin framework) for trainees to rely on as they make sense of—and take action amidst—the heightened uncertainty they will encounter in their clinical practice.

For simple and complex contexts, where patterns repeat, where events are consistent, and where cause‐and‐effect relationships are evident and/or discoverable, practitioners should be able to navigate clinical problems through fact‐based management.^18^ For complex and chaotic contexts, however, entirely different approaches to problem‐solving will be needed to emerge from the uncertainty presented in the clinical environment. The early stages of the pandemic were complex: right answers to management were not readily available; instructive patterns were emergent; leadership was pattern‐based; and creative and innovative approaches were required. To be prepared for complex situations in the clinical environment, HSS can equip learners with skills in problem‐solving and sense‐making, such as being able to pose higher‐order, divergent questions to their teams during times of uncertainty, or leading conversations that can help generate ideas and creative solutions. The foundational domains of HSS themselves (ie, leadership, teaming, ethics, and change agency, management, and advocacy) can also serve as tools to manage and navigate uncertainty in clinical practice.

Conceptually, training in health professions education should highlight the uncertainty that is intrinsic to clinical practice and openly embrace it, rather than trying to eliminate it in curricula. Frameworks borrowed from complexity science can serve as tools to help clinicians better define uncertainty, better identify strategies to deal with this uncertainty in the clinical environment, and avoid using reductionist approaches to complex situations.^28^ Similarly, we have opportunities to leverage the humanities, as well as innovative problem‐solving in formal curricula to better prepare trainees for the complexity and uncertainty that lie ahead. Educators and curriculum designers must also ensure that the educational innovation that was witnessed during the pandemic continues to thrive, and does not revert back to the pre‐COVID status quo.^15^ Reflecting on lessons learned from the pandemic, a mandate to longitudinally include HSS in formal curriculum is required to better prepare medical school graduates to address the complexities and the uncertainty that lies ahead in their professional careers.

## CONFLICT OF INTEREST

Not applicable.

## AUTHOR CONTRIBUTIONS

Conceptualization: Dimitrios Papanagnou, Rebecca Jaffe

Supervision: Dimitrios Papanagnou, Deborah Ziring

Writing—Original Draft Preparation: Dimitrios Papanagnou

Writing—Review & Editing: Rebecca Jaffe, Deborah Ziring, Dimitrios Papanagnou

All authors have read and approved the final version of the manuscript.

## ETHICS STATEMENT

Not applicable.

## Data Availability

Data sharing is not applicable to this article as no new data were created or analyzed in this study.

## References

[1] Cooke S , Lemay J‐F . Transforming medical assessment: integrating uncertainty into the evaluation of clinical reasoning in medical education. Acad Med. 2017;92(6):746‐751. 28557933 10.1097/ACM.0000000000001559

[2] Tonelli MR , Upshur REG . A philosophical approach to addressing uncertainty in medical education. Acad Med. 2019;94(4):507‐511. 30379664 10.1097/ACM.0000000000002512

[3] Lee C , Hall K , Anakin M , Pinnock R . Towards a new understanding of uncertainty in medical education. J Eval Clin Pract. 2020;21:2. 10.1111/jep.13503 33089607

[4] Wasfy JH . Learning about clinical uncertainty. Acad Med. 2006;81(12):1075. 17122472 10.1097/01.ACM.0000242576.25803.65

[5] Hojat M , Vergare MJ , Maxwell K , et al. The devil is in the third year: a longitudinal study of erosion of empathy in medical school. Acad Med. 2009;84(9):1182‐1191. 19707055 10.1097/ACM.0b013e3181b17e55

[6] Leung J , Cloninger CR , Hong BA , Cloninger KM , Eley DS . Temperament and character profiles of medical students associated with tolerance of ambiguity and perfectionism. PeerJ. 2019;7:e7109. 31223537 10.7717/peerj.7109 PMC6571128

[7] Allison JJ , Kiefe CI , Cook EF , Gerrity MS , Orav EJ , Centor R . The association of physician attitudes about uncertainty and risk taking with resource use in a Medicare HMO. Med Decis Making. 1998;18(3):320‐329. 9679997 10.1177/0272989X9801800310

[8] Innes SI , Leboeuf‐Yde C , Walker BF . The relationship between intolerance of uncertainty in chiropractic students and their treatment intervention choices. Chiropr Man Therap. 2017;25:20. 10.1186/s12998-017-0150-2 PMC5518163 28815014

[9] Merrill JM , Camacho Z , Laux LF , Lorimor R , Thornby JI , Vallbona C . Uncertainties and ambiguities: measuring how medical students cope. Med Educ. 1994;28(4):316‐322. 7862004 10.1111/j.1365-2923.1994.tb02719.x

[10] Kim K , Lee Y‐M . Understanding uncertainty in medicine: concepts and implications in medical education. Korean J Med Educ. 2018;30(3):181‐188. 30180505 10.3946/kjme.2018.92 PMC6127608

[11] Walker DHT , Davis PR , Stevenson A . Coping with uncertainty and ambiguity through team collaboration in infrastructure projects. Int J Proj Manage. 2017;35(2):180‐190.

[12] Kocher R , Emanuel EJ , DeParle N‐AM . The affordable care act and the future of clinical medicine: the opportunities and challenges. Ann Intern Med. 2010;153(8):536‐539. 20733178 10.7326/0003-4819-153-8-201010190-00274

[13] Jaffe RC , Bergin CR , Loo LK , et al. Nested domains: a global conceptual model for optimizing the clinical learning environment. Am J Med. 2019;132(7):886‐891. 30953633 10.1016/j.amjmed.2019.03.019

[14] Gruppen LD , Irby DM , Durning SJ , Maggio LA . Conceptualizing learning environments in the health professions. Acad Med. 2019;94(7):969‐974. 30870148 10.1097/ACM.0000000000002702

[15] Thakur A , Soklaridis S , Crawford A , Mulsant B , Sockalingam S . Using rapid design thinking to overcome COVID‐19 challenges in medical education. Acad Med. 2021;96(1):56‐61. 32889947 10.1097/ACM.0000000000003718 PMC7489231

[16] Murugan S , Rajavel S , Aggarwal A , Singh A . Volatility, uncertainty, complexity and ambiguity (VUCA) in context of the COVID‐19 pandemic: challenges and way forward. Int J Health Syst Implement Res. 2020;4(2):10‐16.

[17] Gonzalo JD , Hammoud MM , Starr SR . Chapter 2: systems thinking in healthcare: addressing the complex dynamics of patients and health systems. In: Skochelak SE , Hammoud MM , Lomis KD , et al., eds. AMA Medical Education Consortium: Health Systems Science. 2nd ed. Philadelphia, PA: Elsevier; 2021:21‐36.

[18] Snowden DJ , Boone ME . A leader's framework for decision making. A leader's framework for decision making. Harv Bus Rev. 2007;85(11):68‐76. 149. 18159787

[19] Noyer AL , Esteves JE , Thomson OP . Influence of perceived difficulty of cases on student osteopaths' diagnostic reasoning: a cross sectional study. Chiropr Man Therap. 2017;25:32. 10.1186/s12998-017-0161-z PMC5709833 29214014

[20] Cristancho SM , Apramian T , Vanstone M , Lingard L , Ott M , Novick RJ . Understanding clinical uncertainty: what is going on when experienced surgeons are not sure what to do? Acad Med. 2013;88(10):1516‐1521. 23969352 10.1097/ACM.0b013e3182a3116f PMC5578757

[21] Kumagai AK . A conceptual framework for the use of illness narratives in medical education. Acad Med. 2008;83(7):653‐658. 18580082 10.1097/ACM.0b013e3181782e17

[22] Rising KL , Papanagnou D , McCarthy D , Gentsch A , Powell R . Emergency medicine resident perceptions about the need for increased training in communicating diagnostic uncertainty. Cureus. 2018;10(1):e2088. 29564193 10.7759/cureus.2088 PMC5858850

[23] Rising KL , Powell RE , Cameron KA , et al. Development of the uncertainty communication checklist: a patient‐centered approach to patient discharge from the emergency department. Acad Med. 2020;95(7):1026‐1034. 32101919 10.1097/ACM.0000000000003231 PMC7302334

[24] Papanagnou D , Klein MR , Zhang XC , et al. Developing standardized patient‐based cases for communication training: lessons learned from training residents to communicate diagnostic uncertainty. Adv Simul. 2021;6(1):26. 10.1186/s41077-021-00176-y PMC8296470 34294153

[25] Gonzalo JD , Ogrinc G . Health systems science: the “broccoli” of undergraduate medical education. Acad Med. 2019;94(10):1425‐1432. 31149925 10.1097/ACM.0000000000002815

[26] Ilgen JS , Eva KW , de Bruin A , Cook DA , Regehr G . Comfort with uncertainty: reframing our conceptions of how clinicians navigate complex clinical situations. Adv Health Sci Educ Theory Pract. 2019;24(4):797‐809. 30390181 10.1007/s10459-018-9859-5

[27] Gheihman G , Johnson M , Simpkin AL . Twelve tips for thriving in the face of clinical uncertainty. Med Teach. 2020;42(5):493‐499. 30912996 10.1080/0142159X.2019.1579308

[28] Van Beurden EK , Kia AM , Zask A , Dietrich U , Rose L . Making sense in a complex landscape: how the Cynefin framework from complex adaptive systems theory can inform health promotion practice. Health Promot Int. 2013 Mar;28(1):73‐83. 22128193 10.1093/heapro/dar089

